# Supplementary material for: Longitudinal analysis of immune responses to SARS-CoV-2 recombinant vaccine S-268019-b in phase 1/2 prime-boost study
Source: Front Immunol. 2025 Mar 5;16:1550279. doi: 10.3389/fimmu.2025.1550279 (PMC11919840; doi:10.3389/fimmu.2025.1550279)
Supplement: Supplementary file 1 [file DataSheet1.docx]

**SUPPLEMENTARY MATERIAL**

**Figure S1. Immunogenicity of S-268019-b (5 μg and placebo).**

**(A)** and **(B)** GMT for anti-spike protein IgG antibody, in participants receiving 5 μg S‑268019‑b and placebo. **(C)** and **(D)** GMT for SARS‑CoV‑2 neutralizing antibody, in participants receiving 5 μg S‑268019‑b and placebo. **(E)** Percentage of T cells which produces IFN-γ or IL-2 (Th1) cells and IL- 4 or IL-5 (Th2) cells among CD4^+^ T cells by time point. Statistical analyses were performed using pairwise Wilcoxon signed rank test with multiple comparison correction for (e) (**P*<0.05, ***P*<0.005, ****P*<0.0005).


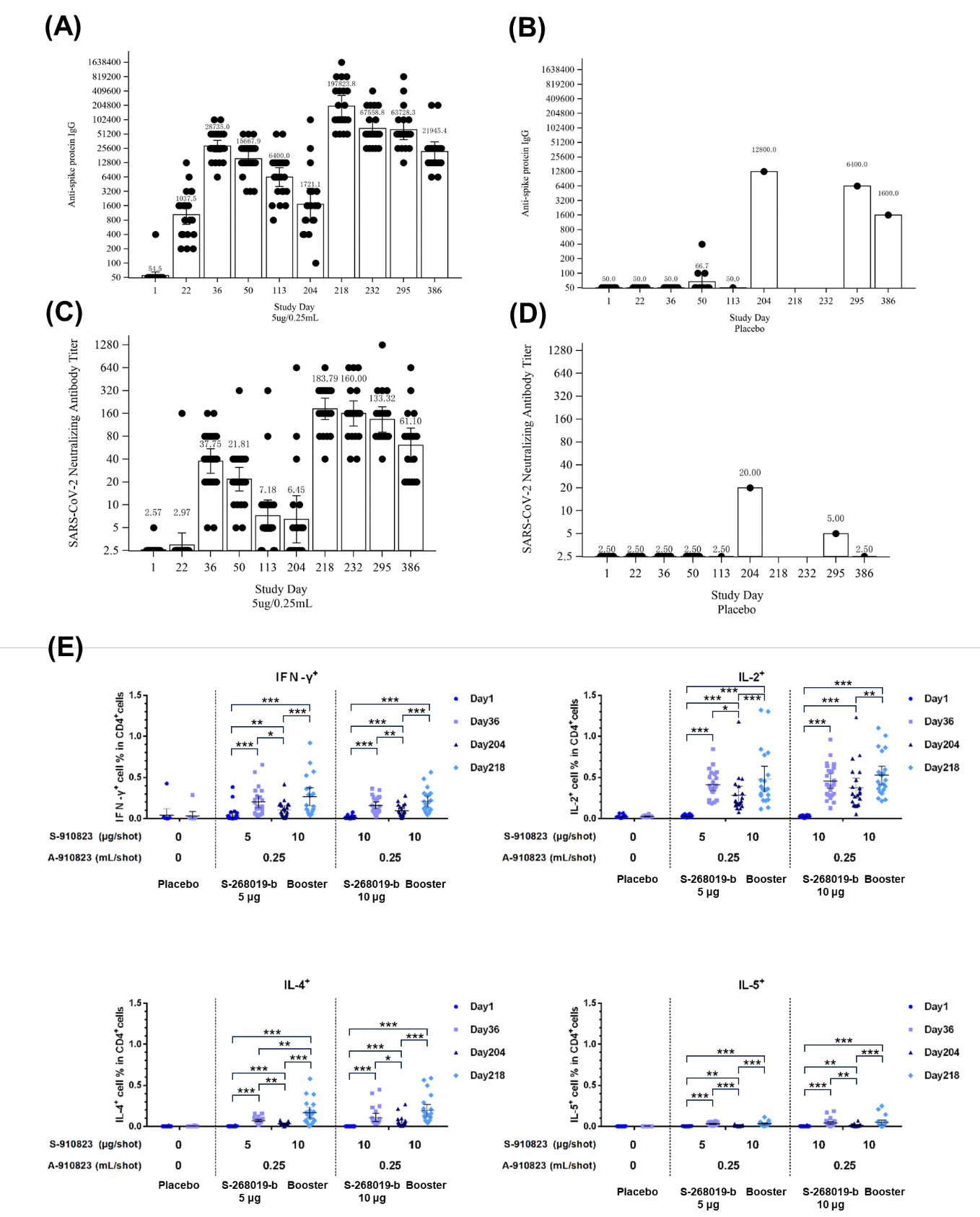


Group = Actual intervention in the primary series. Group names show the antigen content (μg)/adjuvant content (mL).

Abbreviations: GMT, geometric mean titers; IgG, immunoglobulin G; SARS‑CoV‑2, severe acute respiratory syndrome coronavirus 2; IFN-γ, interferon gamma; IL, interleukin.

**Figure S2. Probe validation and gating strategy of B cells.**

**(A**) Confirmation of antigen probe performance to distinguish B cells from other cell types. **(B)** Sorting strategy employed to isolate fraction of B cells. The APC^+^PE^+^ fraction was selected for further analysis.

**
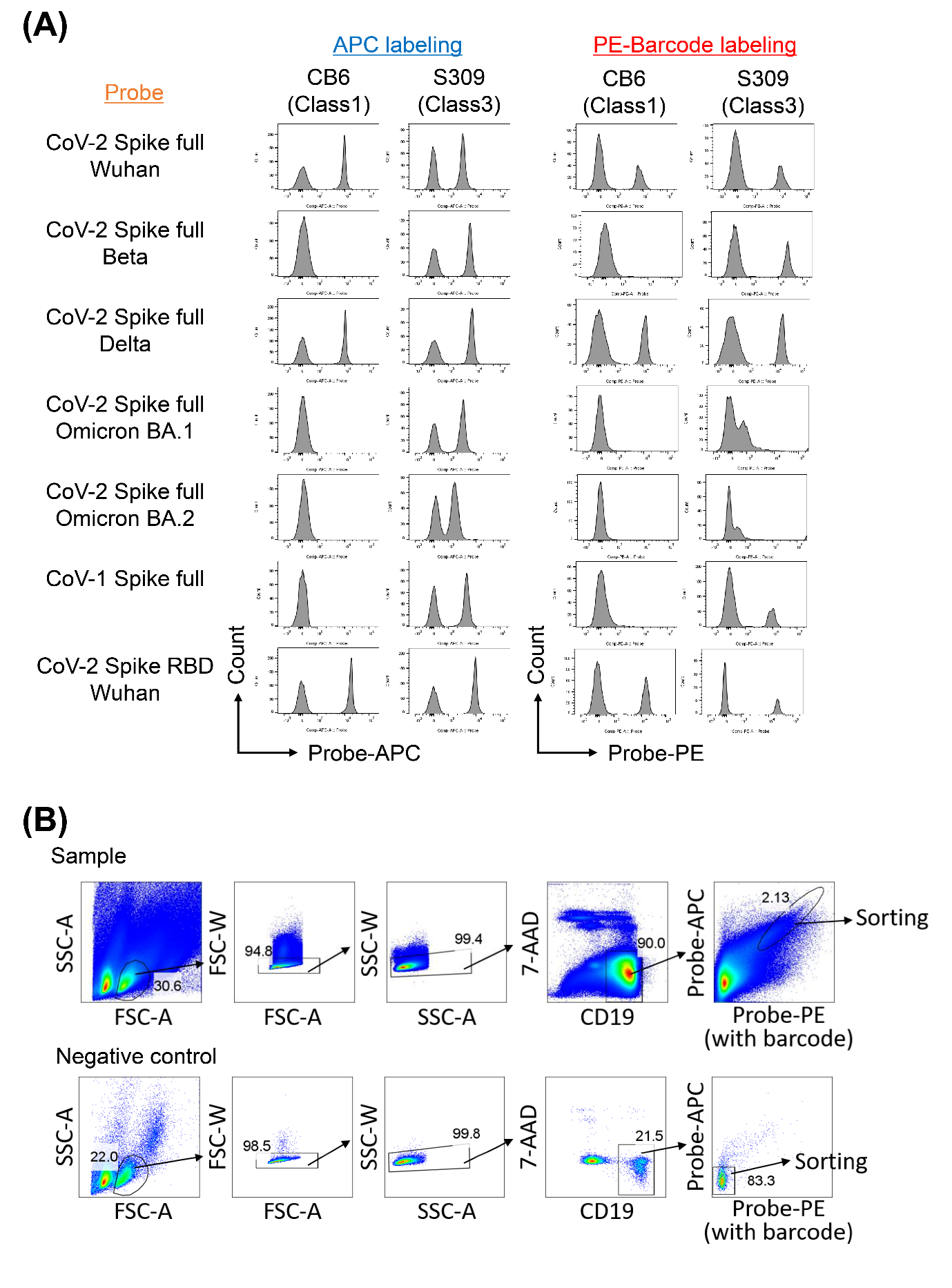
**

**Figure S3. Single-cell analysis of memory B cells (additional data).**

**(A)** Bar graphs depicting the distribution of different memory B-cell subtypes across various time points. Each bar represents mean ± SD. **(B)** Vector field plots illustrating RNA velocity within single-cell transcriptomics data. **(C)** Isotype distribution (e.g., IgG, IgA, IgM) produced by memory B cells at various time points. **(D)** Distribution of heavy chain isotype and SHM number when the antibodies derived from the two infected individuals were excluded. (Left) Fraction of class-switched cells (IgG, IgA, and IgE) per donor. Each point represents the value for each donor. The connected lines indicate samples derived from the same donor. Each bar represents mean ± SD. (Right) Box plot of SHMs in antigen-specific B cells. **(E)** Graphical representation of the clonal diversity of memory B cells over time. Each point represents the value for each cell. Each bar represents mean ± SD. **(F)** The top panel shows the frequency of different heavy chain variable (V) genes used by memory B cells. The bottom panel provides detailed information on the top 20 most frequently used V genes, including their relative abundances. **(G)** Correlation between Day 50 subtype ratio and neutralizing antibody titer. Statistical analyses were performed using pairwise Wilcoxon signed rank test with multiple comparison correction for (a) and (e) and Spearman’s rank-order correlation test for (g) (**P*<0.05, ***P*<0.005, ****P*<0.0005).


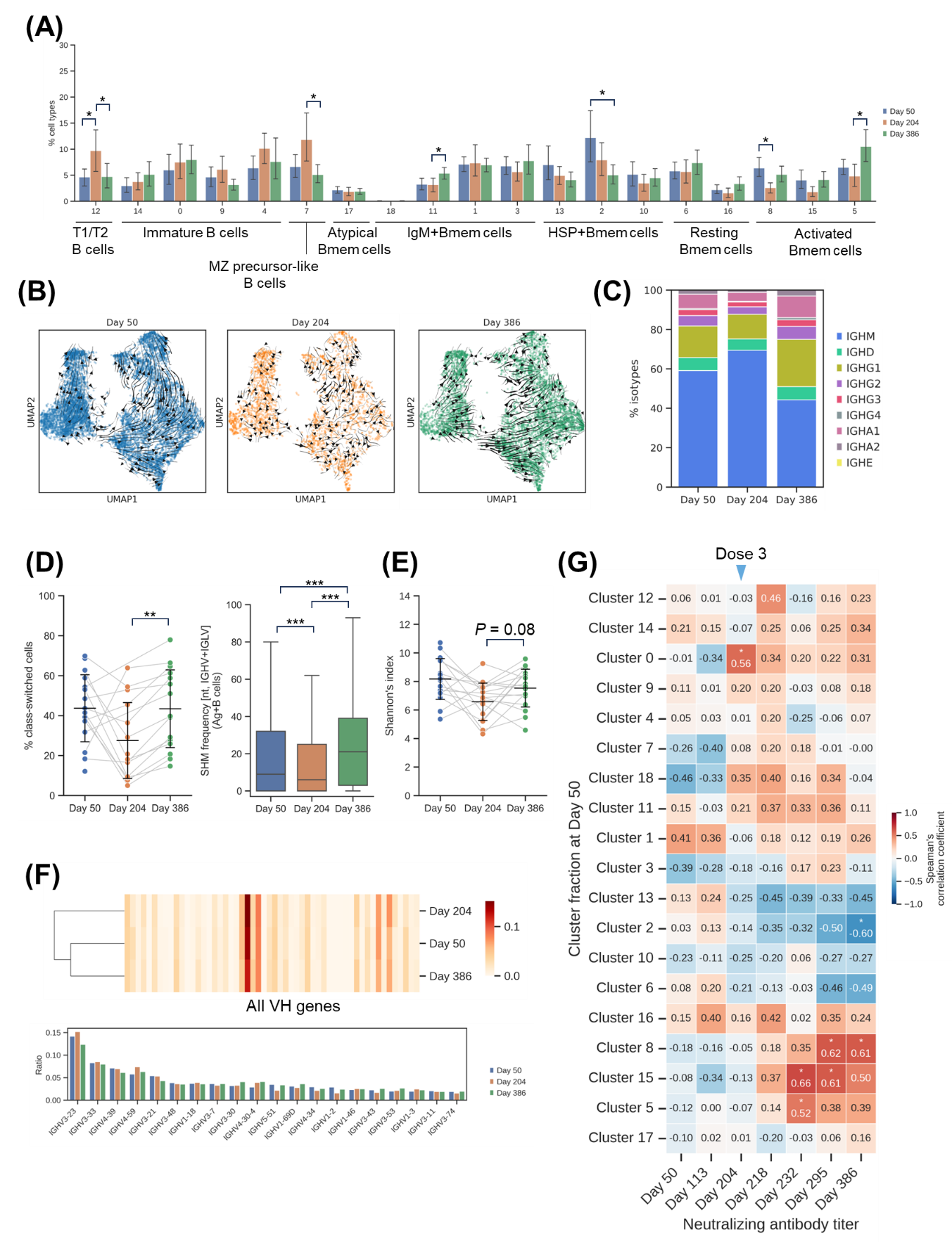


**Figure S4. Epitope mapping of memory B cells (additional data).**

**(A)** Summary pie chart presenting ELISA results and pseudovirus assay results when the antibodies derived from the two infected individuals were excluded. The numbers within the circles represent the number of antibodies. (Left) The distribution of antigen-binding antibodies is determined based on ELISA results. Antibodies were categorized as follows based on the number of strains with titers of 100 ng/mL or below: cross-reactive: two or more strains, strain-specific: only one strain, weak/not-binding: none of the strains. (Right) The distribution of neutralizing antibodies determined based on pseudovirus assay results. The IC50 threshold for categorization is 1000 ng/mL. **(B)** A summary of memory B-cell clones that are shared across different time points. **(C)** Representative examples of each class of identified epitopes. **(D)** A detailed summary of the class 3_1 antibodies, including the sequences of the RBD they recognize, the specific epitopes targeted, results from functional assays, and their mapping on the 3D structure of the protein. Statistical analyses were performed using Fisher’s exact test with multiple comparison correction for (c) (**P*<0.05, ***P*<0.005).

**
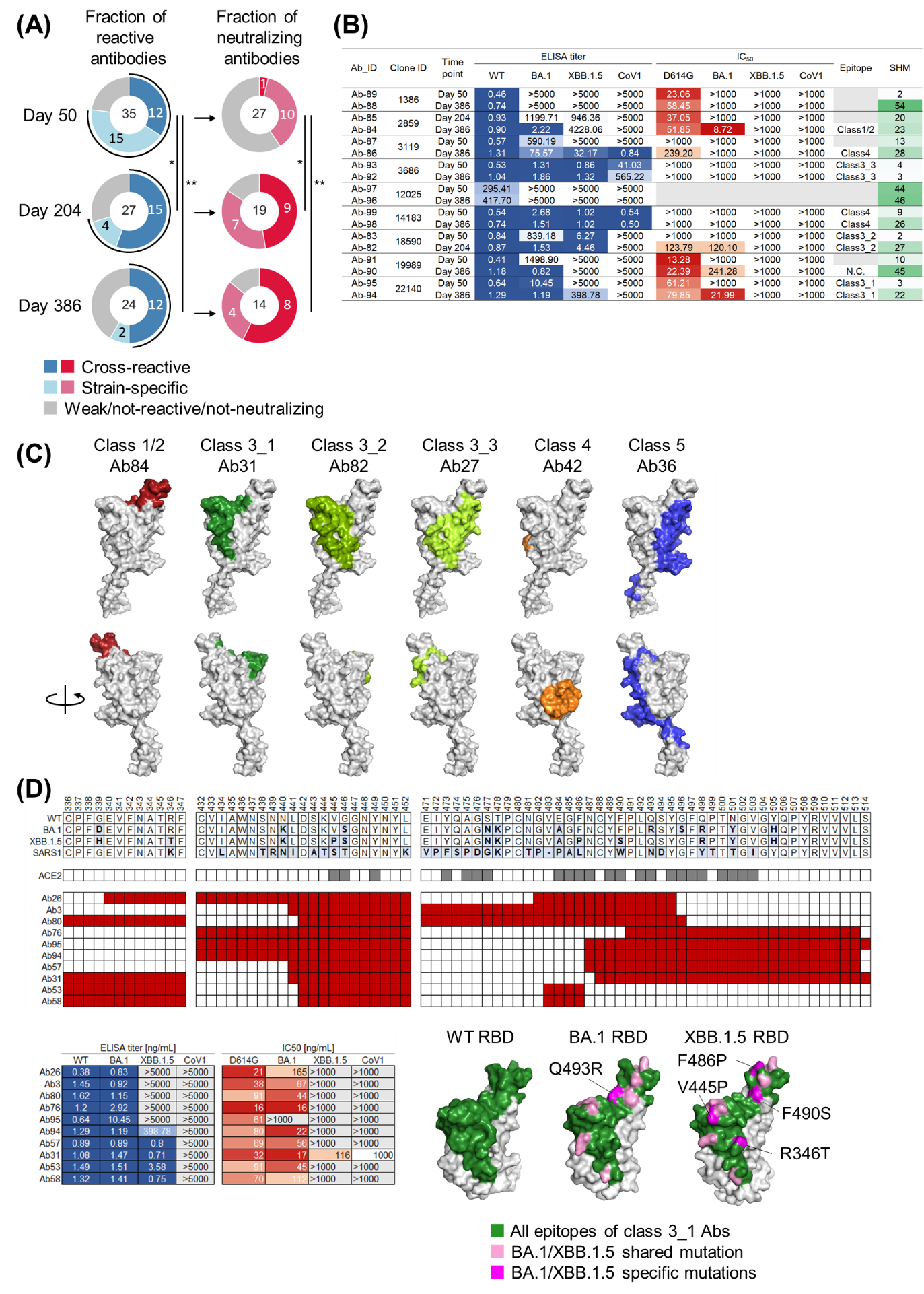
**

**Figure S5. Single-cell analysis of T cells after priming vaccination (additional data).**

**(A)** Dot plots illustrate the gating strategy employed to isolate this specific fraction of T cells, CD3^+^CTV^−^ fraction used for analysis. **(B)** The fraction of T-cell subtype. Each point represents the value for each donor. The connected lines indicate samples derived from the same donor. Each bar represents mean ± SD. **(C)** Scatter plot showing the percentage of bulk T-cell data that corresponds to clones with identified epitopes. **(D)** Flow diagram detailing the process of aggregating public clones. **(E)** UMAP plot showing the distribution of public clone T cells. **(F)** Chart indicating the distribution of public clone T cells among different donors. **(G)** Information on the epitopes recognized by the public T-cell clones. Statistical analyses were performed using pairwise Wilcoxon signed rank test with multiple comparison correction for (b) (**P*<0.05).


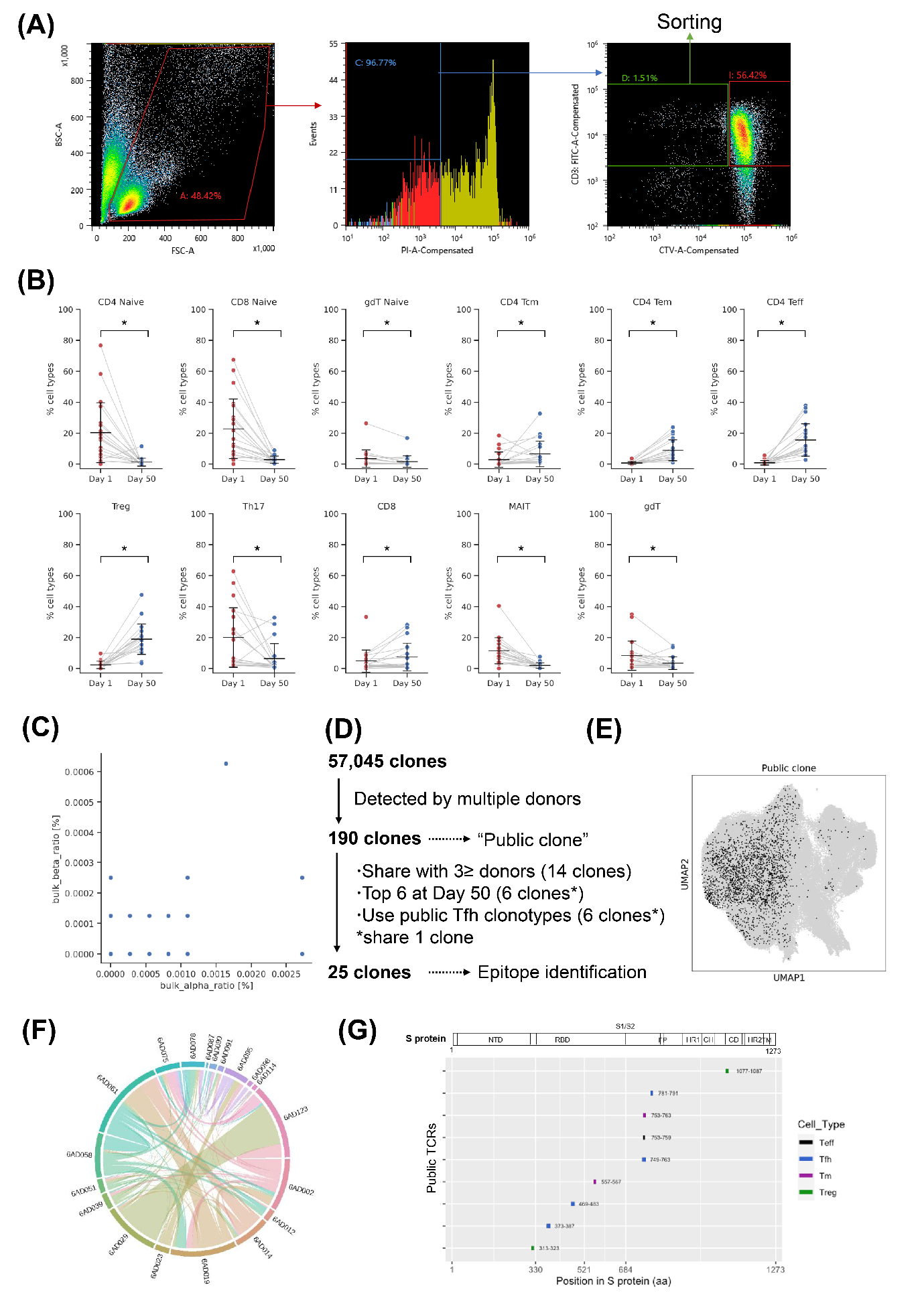


Abbreviations: Tfh, T follicular helper cells; Teff, effector T cells; Tm, memory T; Treg, T regulatory cells

**Figure S6. Single-cell analysis of T cells after booster vaccination (additional data).**

**(A)** Bar graph showing the frequency of different T-cell subtypes at each time point. **(B)** Bar graphs showing the ratio of different T-cell subtypes at each time point. Each point represents the value for each donor. The connected lines indicate samples derived from the same donor. Each bar represents mean ± SD. **(C)** Bar graphs showing the TCR clonotype, representing the temporal expression patterns of TCRs in response to booster vaccination. **(D)** Graphs presenting the Shannon diversity index for each T-cell subtype. Each bar represents mean ± SD. Statistical analyses were performed using pairwise Wilcoxon signed rank test with multiple comparison correction for (b) and (d) (**P*<0.05, ***P*<0.005, ****P*<0.0005).


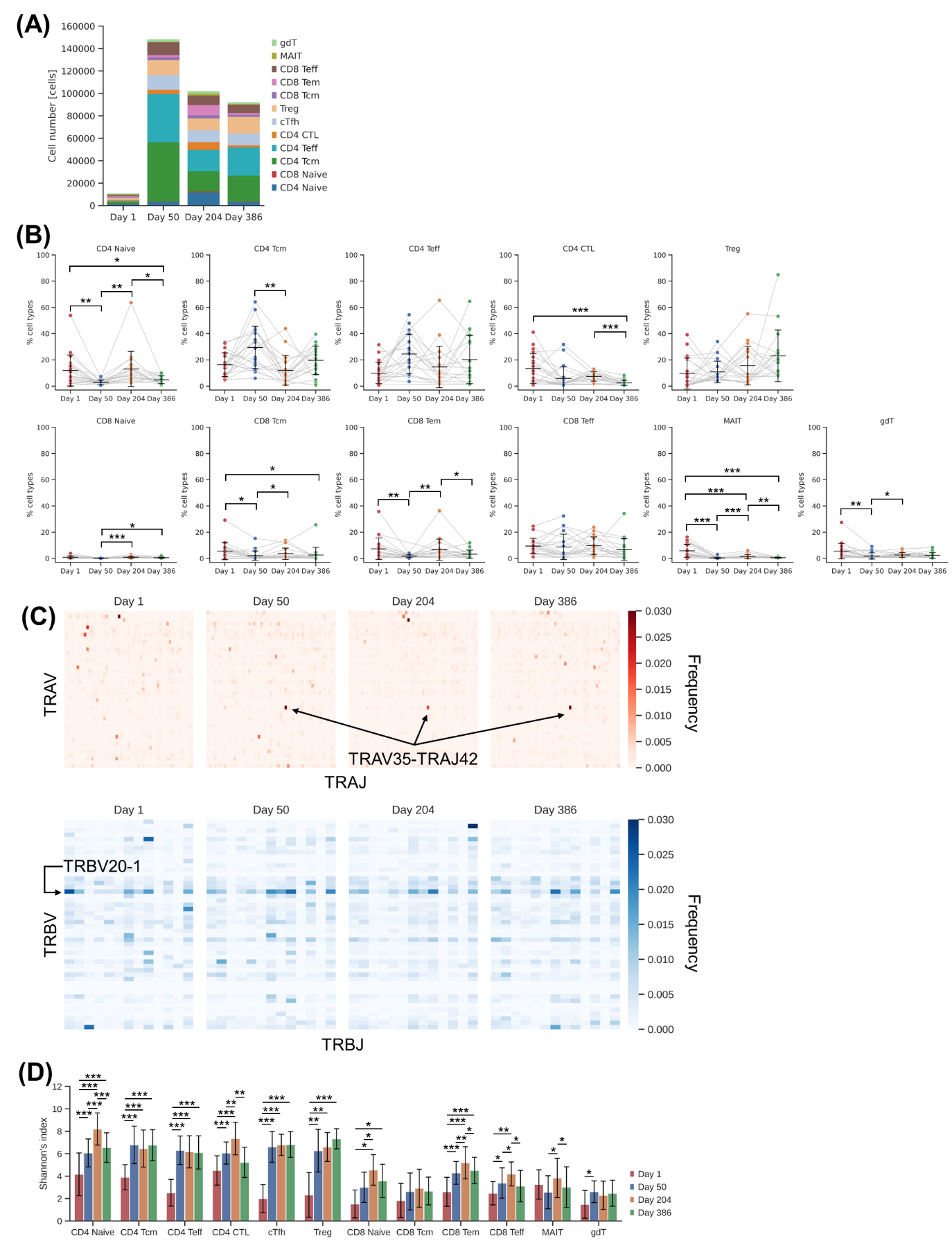


**Table S1. Definition of RBD epitope class.**

| **Epitope class** | **Residue numbers** | **Reference** |
| --- | --- | --- |
| Class 1/2 | 415-421, 452-457, 473-478, 483-494 | (1,2) |
| Class 3 | 333-361, 439-451, 498-501 | (1–5) |
| Class 3_1 | 336-347, 441-452, 487-514 | (1–5) |
| Class 3_2 | 336-361, 441-452 | (1–5) |
| Class 3_3 | 336-361, 453-472 | (1–5) |
| Class 4 | 364-386, 405-408, 412-414, 426-430, 502-504, 515-517 | (2,6) |
| Class 5 | 393-396, 426-430, 462-466, 514-521 | (4) |
| Not classified | 362-363, 387-392, 397-404, 409-411, 422-425, 431-438, 458-461, 467-472, 479-482, 495-497, 505-513, 522-537 |  |

**Table S2. Geometric mean fold rises of anti-spike protein IgG and SARS-CoV-2 neutralizing antibody titers by time point.**

| **Time Point** | **Statistic** | **Anti-spike protein IgG** | | | **SARS-CoV-2 neutralizing antibody titers** | | |
| --- | --- | --- | --- | --- | --- | --- | --- |
|  |  | **5 μg/0.25 mL** | **10 μg/0.25 mL** | **Placebo** | **5 μg/0.25 mL** | **10 μg/0.25 mL** | **Placebo** |
|  |  | ***N* = 24** | ***N* = 24** | ***N* = 12** | ***N* = 24** | ***N* = 24** | ***N* = 12** |
| Day 22 | *n* | 24 | 24 | 12 | 24 | 24 | 12 |
|  | GMFR (95% CI) | 19.03 (12.66–28.59) | 22.63 (14.96–34.23) | 1.00 (–) | 1.16 (0.86–1.56) | 1.03 (0.97–1.09) | 1.00 (–) |
| Day 36 | *n* | 24 | 24 | 12 | 24 | 24 | 12 |
|  | GMFR (95% CI) | 527.00 (375.27–740.10) | 767.13 (555.95–1058.53) | 1.00 (–) | 14.67 (10.25–21.01) | 18.49 (13.89–24.61) | 1.00 (–) |
| Day 50 | *n* | 24 | 24 | 12 | 24 | 24 | 12 |
|  | GMFR (95% CI) | 287.35 (206.47–399.91) | 469.51 (351.21–627.65) | 1.33 (0.90–1.98) | 8.48 (6.14–11.70) | 11.31 (8.61–14.86) | 1.00 (–) |
| Day 113 | *n* | 23 | 22 | 1 | 23 | 22 | 1 |
|  | GMFR (95% CI) | 116.94 (77.82–175.71) | 159.59 (108.71–234.28) | 1.00 (–) | 2.79 (1.81–4.29) | 3.21 (2.44–4.22) | 1.00 (–) |
| Day 204 | *n* | 19 | 22 | 1 | 19 | 22 | 1 |
|  | GMFR (95% CI) | 30.85 (15.19–62.66) | 34.08 (21.43–54.19) | 256.00 (–) | 2.49 (1.25–4.95) | 2.13 (1.40–3.25) | 8.00 (–) |
| Day 218 | *n* | 20 | 22 | 0 | 20 | 22 | 0 |
|  | GMFR (95% CI) | 3565.78 (1937.30–6563.14) | 2896.31 (2092.45–4008.99) | – | 71.01 (51.54–97.85) | 74.92 (58.36–96.17) | – |
| Day 232 | *n* | 20 | 22 | 0 | 20 | 22 | 0 |
|  | GMFR (95% CI) | 1217.75 (736.10–2014.54) | 1237.08 (977.21–1566.06) | – | 61.82 (42.01–90.97) | 66.05 (52.90–82.46) | – |
| Day 295 | *n* | 19 | 22 | 1 | 19 | 22 | 1 |
|  | GMFR (95% CI) | 1142.43 (660.09–1977.24) | 1317.54 (1015.36–1709.66) | 128.00 (–) | 51.42 (34.93–75.69) | 51.33 (41.19–63.97) | 2.00 (–) |
| Day 386 | *n* | 18 | 20 | 1 | 18 | 20 | 1 |
|  | GMFR (95% CI) | 391.02 (224.19–682.02) | 588.13 (388.09–891.28) | 32.00 (–) | 23.52 (14.20–38.95) | 32.00 (21.29–48.10) | 1.00 (–) |

Group = Actual intervention in the primary series. Group names show the antigen content (μg)/adjuvant content (mL).

Abbreviations: CI, confidence interval, GMFR, geometric mean fold rise; IgG, immunoglobulin G; SARS-CoV-2, severe acute respiratory syndrome coronavirus 2.

**Table S3. Summary of monoclonal antibody assay results at different time points.**

| Time point | Ab_ID | ELISA_WT | ELISA_BA.1 | ELISA_XBB.1.5 | ELISA_CoV1 | NT_D614G | NT_BA.1 | NT_XBB.1.5 | NT_CoV1 | Epitope | SHM | Shared | Cluster | H_V gene | H_J gene | H_CDR3 | L_V gene | L_J gene | L_CDR3 |
| --- | --- | --- | --- | --- | --- | --- | --- | --- | --- | --- | --- | --- | --- | --- | --- | --- | --- | --- | --- |
| Day 50 | Ab-1 | 2968.41 | >5000 | >5000 | >5000 |  |  |  |  |  | 72 | No_shared | 15 | IGHV1-46 | IGHJ4 | CSGGSWVDFNFW | IGLV2-8 | IGLJ1 | CASRAGGSKYVF |
| Day 50 | Ab-2 | 3.46 | >5000 | >5000 | >5000 | >1000 | >1000 | >1000 | >1000 |  | 0 | No_shared | 15 | IGHV3-13 | IGHJ4 | CARGYSGYDWVLDYW | IGKV1D-39 | IGKJ4 | CQQSYSTPPLTF |
| Day 50 | Ab-3 | >5000 | >5000 | >5000 | >5000 |  |  |  |  |  | 4 | No_shared | 5 | IGHV3-33 | IGHJ4 | CARVTSSSWDMTGAGYFDYW | IGKV1-33 | IGKJ3 | CQQYDNLPSFTF |
| Day 50 | Ab-4 | 0.45 | >5000 | >5000 | >5000 | 76.7887 | >1000 | >1000 | >1000 |  | 8 | No_shared | 5 | IGHV3-15 | IGHJ6 | CTTDDRLAYYYYGMDVW | IGKV1-5 | IGKJ2 | CQQYNSYRYTF |
| Day 50 | Ab-5 | >5000 | >5000 | >5000 | >5000 |  |  |  |  |  | 5 | No_shared | 5 | IGHV3-21 | IGHJ4 | CASWGSGSIPFDYW | IGLV2-14 | IGLJ3 | CSSYTSSSTWVF |
| Day 50 | Ab-6 | 1.33 | 7.64 | 3.72 | 1.81 | >1000 | >1000 | >1000 | >1000 | NC | 0 | No_shared | 5 | IGHV3-43 | IGHJ3 | CAKVGSVTTFLKGAFDIW | IGKV3-15 | IGKJ5 | CQQYNNWPPGITF |
| Day 50 | Ab-7 | >5000 | >5000 | >5000 | >5000 |  |  |  |  |  | 32 | No_shared | 15 | IGHV4-38-2 | IGHJ6 | CARRPVNGWYEEDDGVSVGVW | IGLV2-11 | IGLJ3 | CCSYAGSTWVF |
| Day 50 | Ab-8 | 429.12 | >5000 | >5000 | >5000 |  |  |  |  |  | 7 | No_shared | 5 | IGHV3-33 | IGHJ4 | CARDYGDYVTHFDYW | IGKV1D-39 | IGKJ1 | CQQSYSTPPWTF |
| Day 50 | Ab-9 | >5000 | >5000 | >5000 | >5000 |  |  |  |  |  | 2 | No_shared | 5 | IGHV3-53 | IGHJ4 | CAREGGYCRGGSCVRAYW | IGKV1-33 | IGKJ5 | CQQYDNLPITF |
| Day 50 | Ab-10 | 0.67 | 1.35 | 1.05 | >5000 | >1000 | >1000 | >1000 | >1000 | Class3_3 | 6 | No_shared | 15 | IGHV3-30 | IGHJ4 | CAKDGDFRYPVFDYW | IGKV1D-39 | IGKJ4 | CQQSYSTPGLTF |
| Day 50 | Ab-11 | >5000 | >5000 | >5000 | >5000 |  |  |  |  |  | 8 | No_shared | 15 | IGHV4-39 | IGHJ4 | CARVVRIIAAAGTSRAFDYW | IGLV2-8 | IGLJ2 | CSSYAGSVVF |
| Day 50 | Ab-12 | 0.5 | 0.99 | 0.77 | 0.94 | >1000 | >1000 | >1000 | >1000 | Class4 | 11 | No_shared | 15 | IGHV5-51 | IGHJ4 | CATSGTYYSGIAYW | IGLV6-57 | IGLJ3 | CQSFDSSNPWVF |
| Day 50 | Ab-13 | 0.67 | >5000 | >5000 | >5000 | 96.8314 | >1000 | >1000 | >1000 |  | 5 | No_shared | 5 | IGHV3-20 | IGHJ4 | CASLIGYSDYDYRTRPQGFDYW | IGLV1-51 | IGLJ3 | CGTWDSSLSAGVF |
| Day 50 | Ab-14 | 0.59 | 1.18 | 0.89 | 395.02 | >1000 | >1000 | >1000 | >1000 | Class3_3 | 63 | No_shared | 5 | IGHV3-23 | IGHJ4 | CTKDGQGHQLYGCYFDSW | IGKV3-20 | IGKJ1 | CQQYRQPPSTF |
| Day 50 | Ab-15 | 1.25 | >5000 | >5000 | >5000 | 150.063 | >1000 | >1000 | >1000 |  | 24 | No_shared | 5 | IGHV1-46 | IGHJ5 | CARDRAQRGYSYPGSW | IGKV1-33 | IGKJ4 | CQQYDNLPLTF |
| Day 50 | Ab-16 | 0.89 | >5000 | 39.32 | 36.98 | >1000 | >1000 | >1000 | >1000 | Class4 | 2 | No_shared | 5 | IGHV4-30-4 | IGHJ5 | CARVVELGIGWFDPW | IGLV3-21 | IGLJ3 | CQVWDSSSDHPVF |
| Day 50 | Ab-17 | 1.69 | >5000 | >5000 | >5000 | >1000 | >1000 | >1000 | >1000 |  | 2 | No_shared | 15 | IGHV5-10-1 | IGHJ4 | CARLNGIATTGLW | IGKV1-5 | IGKJ2 | CQQYNSYF |
| Day 50 | Ab-18 | 0.92 | 1.78 | 1.35 | 110.38 | >1000 | >1000 | >1000 | >1000 | Class5 | 3 | No_shared | 15 | IGHV1-69D | IGHJ4 | CARGPALTKGIAAAGTAFDYW | IGKV4-1 | IGKJ4 | CQQYYSTAPLTF |
| Day 50 | Ab-19 | 0.48 | >5000 | >5000 | >5000 | >1000 | >1000 | >1000 | >1000 |  | 61 | No_shared | 5 | IGHV5-51 | IGHJ3 | CATSARSTRLHPFDVW | IGKV1-5 | IGKJ1 | CQQYSDHWTF |
| Day 50 | Ab-20 | 0.86 | >5000 | >5000 | >5000 | >1000 | >1000 | >1000 | >1000 |  | 2 | No_shared | 5 | IGHV3-48 | IGHJ4 | CARPGRDYYDRSGYYYEEGAFDYW | IGLV3-21 | IGLJ1 | CQVWDSSSDHPGVF |
| Day 50 | Ab-21 | 0.7 | >5000 | >5000 | >5000 | 21.2837 | >1000 | >1000 | >1000 |  | 8 | No_shared | 5 | IGHV4-39 | IGHJ5 | CAREWVVGAIDPYFWFDPW | IGLV1-40 | IGLJ1 | CQSYDTSLSGSKVF |
| Day 50 | Ab-22 | 0.62 | >5000 | >5000 | >5000 | 15.7996 | >1000 | >1000 | >1000 |  | 0 | No_shared | 5 | IGHV3-7 | IGHJ4 | CATLGWLRGFFDYW | IGLV6-57 | IGLJ3 | CQSYDSSNSHWVF |
| Day 50 | Ab-23 | 0.97 | >5000 | >5000 | >5000 | 103.99 | >1000 | >1000 | >1000 |  | 2 | No_shared | 5 | IGHV2-5 | IGHJ6 | CAHSRPWNYYGMDVW | IGLV6-57 | IGLJ2 | CQSYDSSNLVF |
| Day 50 | Ab-24 | 1.14 | >5000 | >5000 | >5000 | >1000 | >1000 | >1000 | >1000 |  | 41 | No_shared | 5 | IGHV1-18 | IGHJ4 | CARMDYYDILTDYQQLWGFDYW | IGLV3-25 | IGLJ1 | CQSADSSGPFYVF |
| Day 50 | Ab-25 | 1.74 | >5000 | >5000 | >5000 | >1000 | >1000 | >1000 | >1000 |  | 1 | No_shared | 5 | IGHV4-30-4 | IGHJ4 | CARVGRGYSYIDYW | IGKV1-33 | IGKJ3 | CQQYDNLLIFTF |
| Day 50 | Ab-26 | 0.38 | 0.83 | >5000 | >5000 | 21.2769 | 164.503 | >1000 | >1000 | Class3_1 | 10 | No_shared | 5 | IGHV3-53 | IGHJ6 | CARDTIYRGMDVW | IGKV1D-39 | IGKJ4 | CQQSYSSLTF |
| Day 50 | Ab-27 | 0.92 | 1.77 | 1.18 | 0.79 | >1000 | >1000 | >1000 | 170.377 | Class3_3 | 3 | No_shared | 15 | IGHV1-2 | IGHJ2 | CARERMTTVTTGLDWYFDLW | IGKV1-27 | IGKJ2 | CQKYNSAPYTF |
| Day 50 | Ab-83 | 0.84 | 839.18 | 6.27 | >5000 | >1000 | >1000 | >1000 | >1000 | Class3_2 | 2 | Two_shared | 15 | IGHV3-21 | IGHJ6 | CARDIRPHLRNSSSWYDYYYYYGMDVW | IGLV1-44 | IGLJ3 | CAAWDDSLNGRVF |
| Day 50 | Ab-87 | 0.57 | 590.19 | >5000 | >5000 | >1000 | >1000 | >1000 | >1000 |  | 13 | Two_shared | 5 | IGHV3-15 | IGHJ3 | CTTAEWLRGAFDIW | IGLV3-21 | IGLJ1 | CQVWDTSSDHVF |
| Day 50 | Ab-89 | 0.46 | >5000 | >5000 | >5000 | 23.0621 | >1000 | >1000 | >1000 |  | 2 | Two_shared | 15 | IGHV3-53 | IGHJ6 | CARDAMTYGMDVW | IGKV1D-39 | IGKJ4 | CQQSYSTLTF |
| Day 50 | Ab-91 | 0.41 | 1498.9 | >5000 | >5000 | 13.2839 | >1000 | >1000 | >1000 |  | 10 | Three_shared | 5 | IGHV1-69D | IGHJ3 | CARQLGRGDYRPSAFDIW | IGKV3-11 | IGKJ3 | CQQPPTF |
| Day 50 | Ab-93 | 0.53 | 1.31 | 0.86 | 41.03 | >1000 | >1000 | >1000 | >1000 | Class3_3 | 4 | Two_shared | 5 | IGHV3-43 | IGHJ5 | CAKDTEQQLLNGANWFDPW | IGLV3-21 | IGLJ3 | CQVWDTFQVF |
| Day 50 | Ab-95 | 0.64 | 10.45 | >5000 | >5000 | 61.2147 | >1000 | >1000 | >1000 | Class3_1 | 3 | Two_shared | 15 | IGHV4-39 | IGHJ4 | CAERILGYCSTTSCYASGYFDYW | IGKV3-15 | IGKJ5 | CQQYNNWPTF |
| Day 50 | Ab-97 | 295.41 | >5000 | >5000 | >5000 |  |  |  |  |  | 44 | Two_shared | 15 | IGHV1-18 | IGHJ5 | CAREKRMIPNWFDPW | IGLV2-11 | IGLJ2 | CCSYAGTYTVVF |
| Day 50 | Ab-99 | 0.54 | 2.68 | 1.02 | 0.54 | >1000 | >1000 | >1000 | >1000 | Class4 | 9 | Two_shared | 5 | IGHV3-53 | IGHJ3 | CARVVSDAFDIW | IGKV1-5 | IGKJ2 | CQQYNAYSYTF |
| Day 204 | Ab-28 | 4160.16 | >5000 | >5000 | 4947.77 |  |  |  |  |  | 11 | No_shared | 5 | IGHV1-69D | IGHJ4 | CARARGYSGYGSLYYFDYW | IGLV1-40 | IGLJ1 | CQSYDSSLSDLVF |
| Day 204 | Ab-30 | 0.49 | 15.04 | 1084.92 | >5000 | 36.8904 | 79.766 | >1000 | 357.577 | Class1/2 | 37 | No_shared | 5 | IGHV4-4 | IGHJ4 | CARTLPPGVVTPHFDHW | IGLV3-19 | IGLJ2 | CNSRDSSGDHLVF |
| Day 204 | Ab-31 | 1.08 | 1.47 | 0.71 | >5000 | 31.8035 | 17.4373 | 115.997 | 1000 | Class3_1 | 34 | No_shared | 5 | IGHV4-39 | IGHJ4 | CARNAWFGELDDYW | IGLV1-47 | IGLJ2 | CATWDDSMSGPVF |
| Day 204 | Ab-32 | 0.59 | 4.51 | 61.94 | >5000 | 28.1158 | >1000 | >1000 | 148.798 | Class1/2 | 29 | Two_shared | 5 | IGHV4-34 | IGHJ4 | CARAAWKTGRFFPDYW | IGKV4-1 | IGKJ1 | CQQYYTSPETF |
| Day 204 | Ab-33 | 0.86 | >5000 | >5000 | >5000 | 103.779 | >1000 | >1000 | 796.784 |  | 26 | No_shared | 5 | IGHV3-48 | IGHJ4 | CARDDYDFSSGYYPPRCDYW | IGLV1-44 | IGLJ2 | CAAWDDSLNVIF |
| Day 204 | Ab-34 | 1.45 | 0.92 | >5000 | >5000 | 37.9474 | 67.252 | >1000 | >1000 | Class3_1 | 36 | No_shared | 5 | IGHV3-49 | IGHJ4 | CSPDGLAVPFW | IGKV1-33 | IGKJ4 | CQQYAKLPLTF |
| Day 204 | Ab-35 | 1.5 | 5.85 | 3.42 | 750.57 | >1000 | >1000 | >1000 | >1000 | NC | 43 | No_shared | 5 | IGHV3-33 | IGHJ5 | CLSDTGGTSAW | IGLV3-10 | IGLJ1 | CYSTDSSGDYSVF |
| Day 204 | Ab-36 | 0.84 | 1.36 | 1.15 | 0.8 | >1000 | >1000 | >1000 | 111.273 | Class5 | 23 | No_shared | 15 | IGHV3-13 | IGHJ2 | CARGYNWNYGWFFDLW | IGKV1-8 | IGKJ5 | CQQSYSSPPVTF |
| Day 204 | Ab-37 | 0.7 | 1.34 | 1120.92 | >5000 | 46.5978 | 8.73251 | >1000 | >1000 | NC | 71 | No_shared | 5 | IGHV4-39 | IGHJ3 | CARRPRGGGDDDPFDVW | IGLV3-21 | IGLJ2 | CQVWDSSSEVVF |
| Day 204 | Ab-38 | 1591.27 | >5000 | >5000 | >5000 |  |  |  |  |  | 27 | No_shared | 5 | IGHV6-1 | IGHJ5 | CARDLAPFGVEVWFDPW | IGKV3-15 | IGKJ2 | CQQYGSWPPTYTF |
| Day 204 | Ab-39 | 4110.63 | >5000 | >5000 | >5000 |  |  |  |  |  | 36 | No_shared | 5 | IGHV3-53 | IGHJ3 | CARDRGEDTFDIW | IGKV1-9 | IGKJ1 | CQQLNANPPTF |
| Day 204 | Ab-40 | 0.69 | >5000 | >5000 | >5000 | 42.3825 | >1000 | >1000 | >1000 |  | 11 | No_shared | 5 | IGHV4-39 | IGHJ5 | CARQNILRFFDPW | IGKV3-15 | IGKJ2 | CQHYNNWYTF |
| Day 204 | Ab-41 | 0.39 | 3.25 | >5000 | >5000 | 215.965 | >1000 | >1000 | >1000 | ND | 29 | No_shared | 5 | IGHV3-33 | IGHJ4 | CARAFMER | IGLV1-40 | IGLJ3 | CQSYDITLSGYMF |
| Day 204 | Ab-42 | 1.38 | 1092.54 | 7.83 | 1.13 | 367.06 | >1000 | >1000 | >1000 | Class4 | 24 | No_shared | 5 | IGHV3-23 | IGHJ1 | CAKDPGVAQQPDYEYFHHW | IGLV3-21 | IGLJ2 | CQVWDSSRDHVVF |
| Day 204 | Ab-43 | >5000 | >5000 | >5000 | >5000 |  |  |  |  |  | 40 | No_shared | 5 | IGHV4-59 | IGHJ5 | CARREWLRGSYDSW | IGLV6-57 | IGLJ2 | CQSYDSGIVAF |
| Day 204 | Ab-44 | 1.58 | 2.77 | 1.97 | 0.98 | >1000 | >1000 | >1000 | >1000 | Class4 | 31 | No_shared | 5 | IGHV1-69D | IGHJ4 | CARQAFRHDDVWGTYRPGAIDYW | IGLV1-44 | IGLJ2 | CATWDDGLNGPVIF |
| Day 204 | Ab-45 | 1.07 | 1.75 | >5000 | >5000 | 207.578 | >1000 | >1000 | >1000 | NC | 29 | No_shared | 5 | IGHV3-21 | IGHJ4 | CARDLMVTSGRVVVRRSFFDSW | IGKV3-11 | IGKJ4 | CQQRNNWPLTF |
| Day 204 | Ab-46 | >5000 | >5000 | >5000 | >5000 |  |  |  |  |  | 30 | Two_shared | 5 | IGHV3-53 | IGHJ4 | CAREAAVATDW | IGKV3-11 | IGKJ4 | CQQRSDWPPLTF |
| Day 204 | Ab-47 | 0.93 | >5000 | >5000 | >5000 | 51.9322 | >1000 | >1000 | >1000 |  | 19 | No_shared | 15 | IGHV3-21 | IGHJ4 | CATDKGPYYDTPWVGW | IGLV4-69 | IGLJ3 | CQTWGTGSWVF |
| Day 204 | Ab-48 | >5000 | >5000 | >5000 | >5000 |  |  |  |  |  | 33 | No_shared | 5 | IGHV1-69D | IGHJ1 | CARGPAYDSGNYYPEYFQHW | IGKV4-1 | IGKJ4 | CHQYYSSPLTF |
| Day 204 | Ab-49 | >5000 | >5000 | >5000 | >5000 |  |  |  |  |  | 34 | No_shared | 5 | IGHV3-30 | IGHJ5 | CARSHFLDENWFDPW | IGKV3-15 | IGKJ3 | CQQYNIWPLVF |
| Day 204 | Ab-50 | 0.88 | 1.35 | 1.13 | 0.8 | >1000 | >1000 | >1000 | 166.852 | Class5 | 46 | Two_shared | 15 | IGHV3-33 | IGHJ3 | CAREGRPNYYDRTYELDTW | IGKV1-5 | IGKJ1 | CQQYNTYRRTF |
| Day 204 | Ab-51 | 0.94 | 1.5 | 1.14 | 1.11 | >1000 | >1000 | >1000 | >1000 | Class3_3 | 64 | No_shared | 5 | IGHV3-23 | IGHJ5 | CAKLLLRSLRNPVDHW | IGKV3-20 | IGKJ1 | CQHYGSSPRSF |
| Day 204 | Ab-52 | 0.73 | 1.39 | 0.92 | 0.85 | >1000 | >1000 | >1000 | >1000 | Class3_3 | 28 | No_shared | 5 | IGHV3-53 | IGHJ3 | CAREVKDAFDIW | IGKV1-9 | IGKJ2 | CQQLNSHPPRGTF |
| Day 204 | Ab-53 | 1.49 | 1.51 | 3.58 | >5000 | 90.5944 | 44.964 | >1000 | >1000 | Class3_1 | 0 | No_shared | 5 | IGHV4-30-4 | IGHJ6 | CARYCSGGRCYALYYYYGMDVW | IGKV3-11 | IGKJ4 | CQQRSNWLTF |
| Day 204 | Ab-54 | 1.12 | 1.9 | 1.17 | 322.89 | 504.835 | 84.3745 | >1000 | >1000 | Class3_3 | 25 | No_shared | 5 | IGHV3-7 | IGHJ4 | CARDRSPLLWFVEAHFDYW | IGLV3-25 | IGLJ2 | CQSGDSSGVVF |
| Day 204 | Ab-82 | 0.87 | 1.53 | 4.46 | >5000 | 123.794 | 120.1 | >1000 | >1000 | Class3_2 | 27 | Two_shared | 5 | IGHV3-21 | IGHJ6 | CARDISPHLRNSSTWYDYYYYDGMDVW | IGLV1-44 | IGLJ3 | CSTWDDTLNGRVF |
| Day 204 | Ab-85 | 0.93 | 1199.71 | 946.36 | >5000 | 37.0547 | >1000 | >1000 | >1000 |  | 20 | Two_shared | 5 | IGHV3-66 | IGHJ3 | CARDLEVVGAFDIW | IGKV3-20 | IGKJ3 | CQQYGTSPPVTF |
| Day 204 | Ab-29 | >5000 | >5000 | >5000 | >5000 |  |  |  |  |  | 32 | No_shared | 5 | IGHV4-4 | IGHJ6 | CASLRKGHIAVPGPFYYYGLDVW | IGLV2-8 | IGLJ1 | CSSYAGDNRNYVF |
| Day 386 | Ab-55 | >5000 | >5000 | >5000 | >5000 |  |  |  |  |  | 24 | No_shared | 5 | IGHV3-43 | IGHJ4 | CAKDFFRLDYHGSGNYEGALDYW | IGKV1-5 | IGKJ5 | CQQYNGYSPITF |
| Day 386 | Ab-56 | 173.82 | 1828.93 | 462.61 | 261.34 |  |  |  |  |  | 45 | No_shared | 5 | IGHV4-59 | IGHJ4 | CARAFGSRGYLGPPDYW | IGKV3-20 | IGKJ4 | CQQYGTSLTF |
| Day 386 | Ab-57 | 0.89 | 0.89 | 0.8 | >5000 | 69.225 | 56.3564 | >1000 | >1000 | Class3_1 | 46 | No_shared | 5 | IGHV3-53 | IGHJ4 | CARGGYQLLRFW | IGKV3-20 | IGKJ4 | CQQYETAPRVTF |
| Day 386 | Ab-58 | 1.32 | 1.41 | 0.75 | >5000 | 69.5343 | 112.083 | >1000 | >1000 | Class3_1 | 6 | Three_shared | 15 | IGHV4-39 | IGHJ4 | CARDEHGDYAVSYW | IGLV1-40 | IGLJ1 | CQSYDSSLSVQYVF |
| Day 386 | Ab-59 | 2641.03 | >5000 | >5000 | >5000 |  |  |  |  |  | 3 | No_shared | 5 | IGHV1-3 | IGHJ3 | CARGGGLLLPLEAFDIW | IGLV3-19 | IGLJ2 | CNSRDSSGNHLVVF |
| Day 386 | Ab-60 | 0.41 | 1138.1 | 454.14 | >5000 | 18.1035 | >1000 | >1000 | >1000 |  | 58 | No_shared | 15 | IGHV3-74 | IGHJ4 | CWGSHSNYFDHW | IGKV4-1 | IGKJ1 | CLQFYGVPPTF |
| Day 386 | Ab-61 | >5000 | >5000 | >5000 | >5000 |  |  |  |  |  | 40 | No_shared | 15 | IGHV3-74 | IGHJ4 | CVNLNCGGDCYSW | IGKV1D-17 | IGKJ5 | CLQFRDFPFTF |
| Day 386 | Ab-62 | 191.56 | 422.72 | 245.75 | 468.58 |  |  |  |  |  | 28 | Two_shared | 15 | IGHV5-51 | IGHJ4 | CARSRSADYFDYW | IGKV1D-13 | IGKJ3 | CQQFKSYPFTF |
| Day 386 | Ab-63 | 0.56 | 3.18 | 2.37 | >5000 | 28.0105 | 124.807 | >1000 | >1000 | Class1/2 | 34 | No_shared | 5 | IGHV1-69D | IGHJ4 | CARALGPLGGGGGECSHACNYLDSW | IGKV3-20 | IGKJ1 | CQQYGYSPWTF |
| Day 386 | Ab-64 | 0.84 | 1.99 | 1.27 | 0.52 | >1000 | >1000 | >1000 | >1000 | NC | 34 | No_shared | 5 | IGHV3-53 | IGHJ6 | CVRDLQEAYGMDIW | IGKV3-20 | IGKJ5 | CQHYGSSSITF |
| Day 386 | Ab-65 | 472.15 | >5000 | >5000 | >5000 |  |  |  |  |  | 33 | No_shared | 5 | IGHV3-48 | IGHJ4 | CARGCRTPRTDQFFDFW | IGKV3-11 | IGKJ5 | CQQRRSWPPITF |
| Day 386 | Ab-66 | 653.85 | >5000 | >5000 | >5000 |  |  |  |  |  | 45 | No_shared | 5 | IGHV1-46 | IGHJ4 | CAKDRDSVYFGSASDYW | IGKV4-1 | IGKJ5 | CQQYSNSPITF |
| Day 386 | Ab-67 | 217.8 | >5000 | >5000 | >5000 |  |  |  |  |  | 43 | No_shared | 5 | IGHV3-53 | IGHJ4 | CAREIFGFYDQW | IGKV1D-39 | IGKJ2 | CQQSYNTLGKYTF |
| Day 386 | Ab-68 | 396.67 | 816.05 | 612.5 | >5000 |  |  |  |  |  | 53 | No_shared | 5 | IGHV1-69D | IGHJ6 | CARDPAHDDGDYLSDFYGMDVW | IGLV5-45 | IGLJ3 | CLIWHNSAWVF |
| Day 386 | Ab-69 | 377.74 | >5000 | >5000 | >5000 |  |  |  |  |  | 30 | No_shared | 5 | IGHV1-69D | IGHJ4 | CARQRGYSGYGAVYYHDSW | IGLV1-40 | IGLJ2 | CQSHDSSLSHVVF |
| Day 386 | Ab-70 | 193.01 | >5000 | >5000 | >5000 |  |  |  |  |  | 48 | No_shared | 5 | IGHV4-39 | IGHJ5 | CAREEPVRGVKNGWFDPW | IGLV3-21 | IGLJ1 | CQVWDAGNDVYVF |
| Day 386 | Ab-71 | 1.08 | 3.26 | 109.13 | 4.09 | >1000 | >1000 | >1000 | >1000 | ND | 48 | No_shared | 5 | IGHV7-4-1 | IGHJ4 | CAREVVGATQGYW | IGLV3-25 | IGLJ1 | CQSGDRIGNYVF |
| Day 386 | Ab-72 | >5000 | >5000 | >5000 | >5000 |  |  |  |  |  | 32 | No_shared | 5 | IGHV3-13 | IGHJ5 | CVRARFSSALLENWFDPW | IGKV1D-39 | IGKJ1 | CQQSYGTPPWTF |
| Day 386 | Ab-73 | 885.97 | >5000 | >5000 | >5000 |  |  |  |  |  | 32 | No_shared | 5 | IGHV1-46 | IGHJ3 | CARGGIVPDVSAAFDIW | IGKV3-20 | IGKJ2 | CQQYGRSAMYTF |
| Day 386 | Ab-74 | 4451.69 | >5000 | >5000 | >5000 |  |  |  |  |  | 30 | No_shared | 5 | IGHV3-53 | IGHJ4 | CTRDFGEFYLDYW | IGKV3-20 | IGKJ2 | CQQYHSSPRTF |
| Day 386 | Ab-75 | 0.96 | 1.17 | 1.12 | 0.53 | >1000 | >1000 | >1000 | 228.752 | Class4 | 38 | No_shared | 15 | IGHV3-43 | IGHJ4 | CTKDMSLTITMAGLFESW | IGKV3-20 | IGKJ1 | CQQYGSSPATF |
| Day 386 | Ab-76 | 1.2 | 2.92 | >5000 | >5000 | 15.5648 | 15.4989 | >1000 | >1000 | Class3_1 | 20 | No_shared | 5 | IGHV1-46 | IGHJ4 | CARGGIVPDVTEPFDYW | IGKV1-5 | IGKJ1 | CQQYNSYSWTF |
| Day 386 | Ab-77 | 39.16 | 40.3 | 31.72 | 142.99 | >1000 | >1000 | >1000 | >1000 | ND | 14 | No_shared | 5 | IGHV3-43 | IGHJ4 | CAKDMFFSVGPTSSGFDYW | IGKV1D-39 | IGKJ2 | CQQSYSSSRTF |
| Day 386 | Ab-78 | >5000 | >5000 | >5000 | >5000 |  |  |  |  |  | 39 | No_shared | 15 | IGHV3-23 | IGHJ2 | CAKDGRGHNRYSPPTNWYFDLW | IGKV4-1 | IGKJ2 | CQQYFSPPPSTF |
| Day 386 | Ab-79 | >5000 | >5000 | >5000 | >5000 |  |  |  |  |  | 45 | No_shared | 5 | IGHV3-30 | IGHJ4 | CAKDGFYDIMTGYPDYW | IGLV1-47 | IGLJ3 | CAAWDDNLNGWVF |
| Day 386 | Ab-80 | 1.62 | 1.15 | >5000 | >5000 | 91.3557 | 44.1915 | >1000 | >1000 | Class3_1 | 40 | No_shared | 5 | IGHV1-69 | IGHJ5 | CARVTLYSAQFGDLSGWLFSW | IGKV4-1 | IGKJ2 | CQQYFYTPESTF |
| Day 386 | Ab-81 | >5000 | >5000 | >5000 | >5000 |  |  |  |  |  | 54 | No_shared | 15 | IGHV2-5 | IGHJ4 | CAHTVRSYGSGHCFNFW | IGLV2-14 | IGLJ2 | CSSFTNINRDFIF |
| Day 386 | Ab-84 | 0.9 | 2.22 | 4228.06 | >5000 | 51.8503 | 8.72319 | >1000 | >1000 | Class1/2 | 23 | Two_shared | 5 | IGHV3-66 | IGHJ3 | CARDLDVAGAFDIW | IGKV3-20 | IGKJ5 | CQLYGSSPITF |
| Day 386 | Ab-86 | 1.31 | 75.57 | 32.17 | 0.84 | 239.199 | >1000 | >1000 | >1000 | Class4 | 28 | Two_shared | 5 | IGHV3-15 | IGHJ3 | CTTSEWLRGAFHIW | IGLV3-21 | IGLJ1 | CQVWDISTDHVF |
| Day 386 | Ab-88 | 0.74 | >5000 | >5000 | >5000 | 58.4521 | >1000 | >1000 | >1000 |  | 54 | Two_shared | 5 | IGHV3-53 | IGHJ6 | CARDAMSYGLDVW | IGKV1D-39 | IGKJ3 | CQQSYDSPPVTF |
| Day 386 | Ab-90 | 1.18 | 0.82 | >5000 | >5000 | 22.3851 | 241.285 | >1000 | >1000 | NC | 45 | Three_shared | 5 | IGHV1-69D | IGHJ3 | CARQLNRGDYRPSAFDIW | IGKV3-11 | IGKJ3 | CQQPPTF |
| Day 386 | Ab-92 | 1.04 | 1.86 | 1.32 | 565.22 | >1000 | >1000 | >1000 | >1000 | Class3_3 | 3 | Two_shared | 15 | IGHV3-43 | IGHJ5 | CAKDTEQQLVNGANWFDPW | IGLV3-21 | IGLJ3 | CQVWDTFQVF |
| Day 386 | Ab-94 | 1.29 | 1.19 | 398.78 | >5000 | 79.854 | 21.993 | >1000 | >1000 | Class3_1 | 22 | Two_shared | 15 | IGHV4-39 | IGHJ4 | CAERQLGYCSTTSCFASGYFDSW | IGKV3-15 | IGKJ5 | CQQYNNWPTF |
| Day 386 | Ab-96 | 417.7 | >5000 | >5000 | >5000 |  |  |  |  |  | 46 | Two_shared | 15 | IGHV1-18 | IGHJ5 | CAREKRMIPNWFDPW | IGLV2-11 | IGLJ2 | CCSYAGTYTVVF |
| Day 386 | Ab-98 | 0.74 | 1.51 | 1.02 | 0.5 | >1000 | >1000 | >1000 | >1000 | Class4 | 26 | Two_shared | 5 | IGHV3-53 | IGHJ3 | CARVVSDAFDIW | IGKV1-5 | IGKJ2 | CQQYDGSSYTF |

**Table S4. TCR epitope, clonotype, and HLA.**

| **Donor ID** | **Rank** | **TRAV** | **CDR3alpha** | **TRAJ** | **TRBV** | **CDR3beta** | **TRBJ** | **Epitope** | **Epitope (location)** | **Restricting HLA** | **Status** |
| --- | --- | --- | --- | --- | --- | --- | --- | --- | --- | --- | --- |
| 2 | 1 | TRAV5 | CAESGGTGRRALTF | TRAJ5 | TRBV25-1 | CASSESPLYNEQFF | TRBJ2-1 | GWTAGAAAYYV | S253,S257 | DRB1*09:01:02 |  |
| 2 | 2 | TRAV3 | CAVRDQAGTALIF | TRAJ15 | TRBV4-2 | CASSPQGAYRASTDTQYF | TRBJ2-3 | TRFASVYAWNR | S341,S345 | DPA1*02:01/02-DPB1*05:01:01 |  |
| 2 | 3 | TRAV23DV6 | CAASREAGGGADGLTF | TRAJ45 | TRBV4-2 | CASSQDLRNLGGRYEQYF | TRBJ2-7 | |  |  | No CD3 expression |
| 2 | 4 | TRAV35 | CAGNFGNEKLTF | TRAJ48 | TRBV20-1 | CSARDPQGRRIQYF | TRBJ2-4 | NCTFEYVSQPF | S161,S165 | DRB4*01:03 |  |
| 2 | 5 | TRAV12-3 | CAMIKAAGNKLTF | TRAJ17 | TRBV20-1 | CSARDPGLAGGPGGNEQFF | TRBJ2-1 | PPAYTNSFTRGVYYP | S25 | DRB1*09:01:02 |  |
| 12 | 1 | TRAV22 | CAVEQGAQKLVF | TRAJ54 | TRBV20-1 | CSARLQPMNTEAFF | TRBJ1-1 | |  |  | Not S |
| 12 | 2 | TRAV8-4 | CAVNEGSYIPTF | TRAJ6 | TRBV19 | CASSTRTDQYF | TRBJ2-4 | |  |  | No CD3 expression |
| 12 | 3 | TRAV12-3 | CAMSRAGSYQLTF | TRAJ28 | TRBV9 | CASSGGLVAGDTQYF | TRBJ2-3 | KTQSLLIVNNATNVV | S113 | DRB1*11:01:01 |  |
| 12 | 4 | TRAV30 | CGTENQAGTALIF | TRAJ15 | TRBV20-1 | CSAGGYGYTF | TRBJ1-2 | RFQTLLALHRS | S233,S237 | DPA1*01:03:01-DPB1*03:01:01 |  |
| 12 | 5 | TRAV12-2 | CAVNNPLRSNYQLIW | TRAJ33 | TRBV20-1 | CSARSGGEGDTQYF | TRBJ2-3 | |  |  | Not S |
| 14 | 1 | TRAV23DV6 | CAAIAYSGAGSYQLTF | TRAJ28 | TRBV7-9 | CASSSRENIQYF | TRBJ2-4 | |  |  | Not S |
| 14 | 2 | TRAV1-1 | CAVRDDTDKLIF | TRAJ34 | TRBV28 | CASSPFDSGRDNEQFF | TRBJ2-1 | |  |  | Not S |
| 14 | 3 | TRAV12-1 | CVVNRGGGYQKVTF | TRAJ13 | TRBV30 | CAWRGVGAEAFF | TRBJ1-1 | LSFELLHAPAT | S509,S513 | DRB1*01:01:01 |  |
| 14 | 4 | TRAV26-1 | CIVRVGLGDDKIIF | TRAJ30 | TRBV20-1 | CSASAASGSHNEQFF | TRBJ2-1 | INITRFQTLLA | S229,S233 | DRB1*04:03:01 |  |
| 14 | 5 | TRAV6 | CALSSGGSYIPTF | TRAJ6 | TRBV6-2 | CASTPGTVNTEAFF | TRBJ1-1 | EIRASANLAAT | S1013,S1017 | DRB4*01:03 |  |
| 19 | 1 | TRAV17 | CATNDYKLSF | TRAJ20 | TRBV5-6 | CASNVWGGTGSTDTQYF | TRBJ2-3 | |  |  | No CD3 expression |
| 19 | 2 | TRAV29DV5 | CAASEIGSARQLTF | TRAJ22 | TRBV2 | CASRRDLPNYGYTF | TRBJ1-2 | RFQTLLALHRS | S233,S237 | DPA1*02:01:01-DPB1*09:01:01 |  |
| 19 | 3 | TRAV12-1 | CVVNRGSNYQLIW | TRAJ33 | TRBV28 | CASSQDSGTYNEQFF | TRBJ2-1 | LLQYGSFCTQL | S749,753 | DRB5*01:01/02 (DRB1*15:01/02 weak) | |
| 19 | 4 | TRAV26-1 | CIVRPSLSSSARQLTF | TRAJ22 | TRBV5-1 | CASSSTPGQNTEAFF | TRBJ1-1 | |  |  | No CD3 expression |
| 19 | 5 | TRAV2 | CAVHNAGNMLTF | TRAJ39 | TRBV7-3 | CASSLAGGLTDTQYF | TRBJ2-3 | SFSTFKCYGVS | S369,S373 | DRB1*15:02:01 |  |
| 23 | 1 | TRAV12-3 | CAMTRGSGTASKLTF | TRAJ44 | TRBV20-1 | CSAISGQGGADTQYF | TRBJ2-3 | HWFVTQRNFYE | S1097,S1101 | DRB1*08:03:02 |  |
| 23 | 2 | TRAV12-3 | CAMSDSGGYQKVTF | TRAJ13 | TRBV29-1 | CSASRQGTRGYTF | TRBJ1-2 | NASVVNIQKEIDRLN | S1173 | DRB1*08:03:02 |  |
| 23 | 3 | TRAV29DV5 | CAASFSGAGSYQLTF | TRAJ28 | TRBV10-3 | CASVGGSGINEQFF | TRBJ2-1 | FNFNGLTGTGVLTES | S541 | DQA1*01:03:01-DQB1*06:01:01 |  |
| 23 | 4 | TRAV26-1 | CIVRVAALIIQGAQKLVF | TRAJ54 | TRBV11-3 | CASSPGTSGRQDNEQFF | TRBJ2-1 | |  |  | No CD3 expression |
| 23 | 5 | TRAV9-2 | CALLYNNNDMRF | TRAJ43 | TRBV19 | CASSEYQETQYF | TRBJ2-5 | YNYLYRLFRKS | S445,S449 | DPA1*02:02:02-DPB1*02:01:02/02:02:01 | |
| 29 | 1 | TRAV14DV4 | CAMGLNRDDKIIF | TRAJ30 | TRBV5-6 | CASSLDFEQYF | TRBJ2-7 | |  |  | Not S |
| 29 | 2 | TRAV8-2 | CVVSTSSGGSYIPTF | TRAJ6 | TRBV30 | CAWSVTSKGQYF | TRBJ2-7 | |  |  | Not S |
| 29 | 3 | TRAV13-2 | CAEKTNRDDKIIF | TRAJ30 | TRBV5-6 | CASSLGETQYF | TRBJ2-5 | |  |  | Not S |
| 29 | 4 | TRAV8-6 | CAVSLGSARQLTF | TRAJ22 | TRBV30 | CAWSVGSRYGYTF | TRBJ1-2 | FIEDLLFNKVT | S813,S817 | DPA1*01:03:01-DPB1*02:01:02/04:02:01 | |
| 29 | 5 | TRAV13-2 | CAEGVIGQGGKLIF | TRAJ23 | TRBV7-6 | CASSLGPANQPQHF | TRBJ1-5 | |  |  | Not S |
| 39 | 1 | TRAV29DV5 | CAASVLNFGNEKLTF | TRAJ48 | TRBV6-6 | CASGDRAEDEKLFF | TRBJ1-4 | |  |  | Not S |
| 39 | 2 | TRAV13-1 | CAASTEGNKLVF | TRAJ47 | TRBV20-1 | CSAPLTGRWYGYTF | TRBJ1-2 | |  |  | Not S |
| 39 | 3 | TRAV12-3 | CAGERRSNYQLIW | TRAJ33 | TRBV19 | CASSISGTEATEAFF | TRBJ1-1 | TRGVYYPDKVFRSSV | S33 | DQA1*03:03:01-DQB1*03:01:01 |  |
| 39 | 4 | TRAV1-1 | CAVGDVTDKLIF | TRAJ34 | TRBV3-1 | CASSPGLAGGPDTQYF | TRBJ2-3 | LVKNKCVNFNF | S529,S533 | DRB3*03:01:01 |  |
| 39 | 5 | TRAV13-1 | CAAKTG | TRAJ4 | TRBV20-1 | CSAPLTGRWYGYTF | TRBJ1-2 | |  |  | No CD3 expression |
| 51 | 1 | TRAV21 | CAGRTSGGGADGLTF | TRAJ45 | TRBV5-1 | CASRGDTSYNEQFF | TRBJ2-1 | |  |  | No CD3 expression |
| 51 | 2 | TRAV26-2 | CILRDGANNNARLMF | TRAJ31 | TRBV29-1 | CSAHLGGNNEQFF | TRBJ2-1 | YNYLYRLFRKS | S445,S449 | DRB1*11:01:01 |  |
| 51 | 3 | TRAV12-3 | CAMSEGWVRAGKSTF | TRAJ27 | TRBV28 | CASSRTGTDEKLFF | TRBJ1-4 | KKFLPFQQFGR | S553,S557 | DRB1*11:01:01 |  |
| 51 | 4 | TRAV29DV5 | CAASRSLTGNQFYF | TRAJ49 | TRBV20-1 | CSARDFQVGSGNTIYF | TRBJ1-3 | |  |  | No CD3 expression |
| 51 | 5 | TRAV12-2 | CAGRLDTGRRALTF | TRAJ5 | TRBV29-1 | CSVGRGPDRTGELFF | TRBJ2-2 | NCTFEYVSQPF | S161,S165 | DRB1*09:01:02 |  |
| 58 | 1 | TRAV29DV5 | CAASVYGNNRLAF | TRAJ7 | TRBV6-1 | CASSEGFSNQPQHF | TRBJ1-5 | LLQYGSF | S745,S749,S753 | DRB1*15:02:01 |  |
| 58 | 2 | TRAV2 | CAVIQGTGGSYIPTF | TRAJ6 | TRBV7-8 | CASSLDGPSITFFF | TRBJ1-3 | |  |  | No CD3 expression |
| 58 | 3 | TRAV9-2 | CALKKNTGNQFYF | TRAJ49 | TRBV12-4 | CASTSRDRGPTGELFF | TRBJ2-2 | NNSYECDIPIGAGIC | S657 | DRB3*01:01:02 |  |
| 58 | 4 | TRAV38-1 | CAFMRPRTYKYIF | TRAJ40 | TRBV30 | CAWSAGFSGLGTDTQYF | TRBJ2-3 | SVYAWNRKRIS | S345,S349 | DRB1*13:01:01 |  |
| 58 | 5 | TRAV13-1 | CAASSQGGSEKLVF | TRAJ57 | TRBV11-1 | CASSPWTSGQETQYF | TRBJ2-5 | IDRLITGRLQSLQTY | S993 | DQA1*01:03:01-DQB1*06:01/03 |  |
| 61 | 1 | TRAV8-4 | CAVSTDTGRRALTF | TRAJ5 | TRBV30 | CAWSGTGGLSRGYTF | TRBJ1-2 | QALNTLVKQLS | S953,S957 | DPA1*02:02:02-DPB1*05:01:01 |  |
| 61 | 2 | TRAV13-1 | CAASISGSARQLTF | TRAJ22 | TRBV4-2 | CASSQERMGGYEQYF | TRBJ2-7 | |  |  | Not S |
| 61 | 3 | TRAV23DV6 | CAATGYSGAGSYQLTF | TRAJ28 | TRBV7-9 | CASTFRENIQYF | TRBJ2-4 | PFQQFGR | S553,S557,S561 | DPA1*02:02:02-DPB1*05:01:01 |  |
| 61 | 4 | TRAV9-2 | CALSVPNTGGFKTIF | TRAJ9 | TRBV5-5 | CASSILTGGEQYF | TRBJ2-7 | |  |  | Not S |
| 61 | 5 | TRAV25 | CAGAGNNRKLIW | TRAJ38 | TRBV19 | CASSTTGAGTDTQYF | TRBJ2-3 | |  |  | Not S |
| 75 | 1 | TRAV12-1 | CAVNSGAGSYQLTF | TRAJ28 | TRBV6-6 | CASSYEINNEQFF | TRBJ2-1 | |  |  | Not S |
| 75 | 2 | TRAV21 | CAVVTGANNLFF | TRAJ36 | TRBV14 | CASSQVFDTQYF | TRBJ2-3 | LLQYGSFCTQL | S749,S753 | DRB1*15:01:01 |  |
| 75 | 3 | TRAV35 | CAGQRNNNDMRF | TRAJ43 | TRBV2 | CASSVERAPYEQYF | TRBJ2-7 | |  |  | Not S |
| 75 | 4 | TRAV17 | CATDNNNARLMF | TRAJ31 | TRBV28 | CASSPDSRAGEQYF | TRBJ2-7 | CSNLLLQYGSFCTQL | S749 | DRB1*15:01:01 |  |
| 75 | 5 | TRAV9-2 | CALTSGGGATNKLIF | TRAJ32 | TRBV2 | CASSETRVSSTGKLFF | TRBJ1-4 | LQSLQTYVTQQLIRA | S1001 | DRB1*15:01:01 |  |
| 78 | 1 | TRAV14DV4 | CAMREGLGGFKTIF | TRAJ9 | TRBV6-5 | CASSYAGRGDGYTF | TRBJ1-2 | |  |  | Not S |
| 78 | 2 | TRAV1-2 | CAVRNAGNMLTF | TRAJ39 | TRBV9 | CASGGANTEAFF | TRBJ1-1 | |  |  | Not S |
| 78 | 3 | TRAV19 | CALSETEEDTGRRALTF | TRAJ5 | TRBV5-1 | CASSVEQGARADTQYF | TRBJ2-3 | |  |  | Not S |
| 78 | 4 | TRAV9-2 | CALSDQWSRDDKIIF | TRAJ30 | TRBV5-4 | CASSLMADTQYF | TRBJ2-3 | |  |  | Not S |
| 78 | 5 | TRAV9-2 | CALSDQWSRDDKIIF | TRAJ30 | TRBV5-6 | CASRGPGETQYF | TRBJ2-5 | |  |  | Not S |
| 87 | 1 | TRAV21 | CAVRPRAGGTSYGKLTF | TRAJ52 | TRBV6-5 | CASRRDRGNNSPLHF | TRBJ1-6 | |  |  | Not S |
| 87 | 2 | TRAV8-2 | CVVRITGFQKLVF | TRAJ8 | TRBV14 | CASSQARTGELFF | TRBJ2-2 | |  |  | Not S |
| 87 | 3 | TRAV1-1 | CAVRGDSGYSTLTF | TRAJ11 | TRBV10-2 | CASSDQQGRGTQGQPQHF | TRBJ1-5 | LLQYGSF | S745,S749,S753 | DRB1*15:02:01 |  |
| 87 | 4 | TRAV22 | CAVEGTGANSKLTF | TRAJ56 | TRBV29-1 | CSGSRGYSYEQYF | TRBJ2-7 | YENQKLIANQFNSAI | S917 | DPA1*01:03:01-DPB1*04:02:01 |  |
| 87 | 5 | TRAV29DV5 | CAASPQGGSEKLVF | TRAJ57 | TRBV27 | CASSLVVGFEGLHF | TRBJ1-6 | |  |  | Not S |
| 90 | 1 | TRAV27 | CAGDWTGNQFYF | TRAJ49 | TRBV7-2 | CASSEGHYSVGYTF | TRBJ1-2 | LVKNKCVNFNF | S529,S533 | DRB3*03:01:01 |  |
| 90 | 2 | TRAV9-2 | CALSFSGSGNTGKLIF | TRAJ37 | TRBV5-6 | CASSPDTGGSGGYTF | TRBJ1-2 | IFGTTLDSKTQ | S101,S105 | DRB1*13:02:01 |  |
| 90 | 3 | TRAV1-1 | CAALTGGGNKLTF | TRAJ10 | TRBV18 | CASSPGQGDTEAFF | TRBJ1-1 | |  |  | Not S |
| 90 | 4 | TRAV13-1 | CAAVIGTSYGKLTF | TRAJ52 | TRBV28 | CASSSYRQGRNTQYF | TRBJ2-3 | KKFLPFQQFGR | S553,S557 | DRB1*13:02:01 |  |
| 90 | 5 | TRAV4 | CLVGDMDSSYKLIF | TRAJ12 | TRBV25-1 | CASSEYRLAVDEKLFF | TRBJ1-4 | |  |  | No CD3 expression |
| 91 | 1 | TRAV9-2 | CALSDQQAGTALIF | TRAJ15 | TRBV5-4 | CASSWAGTLEQYF | TRBJ2-7 | |  |  | No CD3 expression |
| 91 | 2 | TRAV12-3 | CAMSENSGAGSYQLTF | TRAJ28 | TRBV20-1 | CSARRGQLRNEQYF | TRBJ2-7 | QNVLYENQKLIANQF | S913 | DQA1*01:04:01-DQB1*05:03:01 |  |
| 91 | 3 | TRAV8-6 | CAVEIGGGSYIPTF | TRAJ6 | TRBV20-1 | CSAKTTDSYEQYF | TRBJ2-7 | KTQSLLIVNNATNVV | S113 | DRB3*02:02:01 |  |
| 91 | 4 | TRAV13-1 | CASAGGSNYKLTF | TRAJ53 | TRBV20-1 | CSARVGMIDTQYF | TRBJ2-3 | |  |  | Not S |
| 91 | 5 | TRAV9-2 | CALSDPSNNDMRF | TRAJ43 | TRBV6-1 | CASTIADRQQETQYF | TRBJ2-5 | |  |  | Not S |
| 95 | 1 | TRAV6 | CALGSGNTGKLIF | TRAJ37 | TRBV25-1 | CASTTGGLHETQYF | TRBJ2-5 | EIRASANLAAT | S1013,S1017 | DPA1*01:03:01-DPB1*04:01:01 |  |
| 95 | 2 | TRAV35 | CAGLNYGGSQGNLIF | TRAJ42 | TRBV6-1 | CASSRGSFGYTF | TRBJ1-2 | NCTFEYVSQPFLMDL | S165 | DPA1*01:03:01-DPB1*04:01:01 |  |
| 95 | 3 | TRAV13-2 | CAESYSGNTPLVF | TRAJ29 | TRBV12-4 | CASSSRDRDWRF | TRBJ2-3 | YNYLYRLFRKS | S445,S449 | DPA1*01:03:01-DPB1*02:01:02 |  |
| 95 | 4 | TRAV1-1 | CALSYNTDKLIF | TRAJ34 | TRBV3-1 | CASSPGTVSETQYF | TRBJ2-5 | LVKNKCVNFNF | S529,S533 | DRB3*03:01:01 |  |
| 95 | 5 | TRAV12-3 | CAMSPNTGFQKLVF | TRAJ8 | TRBV7-8 | CASSLGGDTQYF | TRBJ2-3 | FVSGNCDVVIG | S1117,S1121 | DRB1*13:02:01 |  |
| 98 | 1 | TRAV3 | CAVRDAVGSYIPTF | TRAJ6 | TRBV5-1 | CASSLQGLNTEAFF | TRBJ1-1 | KKFLPFQQFGRDIAD | S557 | DQA1*01:02:01-DQB1*06:04/118 |  |
| 98 | 2 | TRAV12-3 | CAMGNNAGNMLTF | TRAJ39 | TRBV20-1 | CSARGPSGGAIDTQYF | TRBJ2-3 | |  |  | Not S |
| 98 | 3 | TRAV8-6 | CAVSEASGGSNYKLTF | TRAJ53 | TRBV18 | CASSQGAGGADTQYF | TRBJ2-3 | KLIANQFNSAI | S917,S921 | DRB3*03:01:01 |  |
| 98 | 4 | TRAV13-1 | CAASKWNTDKLIF | TRAJ34 | TRBV5-6 | CASSHTRTGSEELFF | TRBJ2-2 | |  |  | Not S |
| 98 | 5 | TRAV12-1 | CVVNPNNNDMRF | TRAJ43 | TRBV9 | CASSVSGSADTQYF | TRBJ2-3 | |  |  | Not S |
| 114 | 1 | TRAV12-1 | CVVNKNGYGQNFVF | TRAJ26 | TRBV7-2 | CASSLAGGSQYEQYF | TRBJ2-7 | VFAQVKQIYKT | S777,S781 | DRB1*14:06:01 |  |
| 114 | 2 | TRAV8-4 | CAVTTNTGTASKLTF | TRAJ44 | TRBV12-4 | CASSPLNSQGTEAFF | TRBJ1-1 | |  |  | Not S |
| 114 | 3 | TRAV8-6 | CAVSAAVNTGFQKLVF | TRAJ8 | TRBV6-6 | CASSDREDYGYTF | TRBJ1-2 | YNYLYRLFRKS | S445,S449 | DPA1*01:03:01-DPB1*02:01:02 |  |
| 114 | 4 | TRAV24 | CASITGANNLFF | TRAJ36 | TRBV12-4 | CASSLDRPHEQFF | TRBJ2-1 | |  |  | Not S |
| 114 | 5 | TRAV4 | CLVGDIPPGGYQKVTF | TRAJ13 | TRBV24-1 | CATSVGTGGRVNNEQFF | TRBJ2-1 | |  |  | Not S |
| 123 | 1 | TRAV26-1 | CIVRGPSSGSARQLTF | TRAJ22 | TRBV11-2 | CASSLLAGRGDEQYF | TRBJ2-7 | MIAQYTSALLA | S865,S869 | DRB1*15:01:01 |  |
| 123 | 2 | TRAV8-4 | CAVSDLRNNNDMRF | TRAJ43 | TRBV20-1 | CSARRGTEAFF | TRBJ1-1 | |  |  | No CD3 expression |
| 123 | 3 | TRAV12-3 | CAMSTVSGGYNKLIF | TRAJ4 | TRBV12-4 | CASSRSWGASYNSPLHF | TRBJ1-6 | NVTWFHAIHVSGTNG | S61 | DRB1*15:01:01 |  |
| 123 | 4 | TRAV26-1 | CIVRGPTGANNLFF | TRAJ36 | TRBV9 | CASSVVGAPVSTDTQYF | TRBJ2-3 | MIAQYTSALLA | S865,S869 | DRB1*15:01:01 |  |
| 123 | 5 | TRAV13-1 | CAASKGTSGTYKYIF | TRAJ40 | TRBV20-1 | CSARRATEAFF | TRBJ1-1 | LLQYGSF | S745,S749,S753 | DRB1*15:01:01 |  |

**Supplemental references**

1. Hansen J, Baum A, Pascal KE, Russo V, Giordano S, Wloga E, et al. Studies in humanized mice and convalescent humans yield a SARS-CoV-2 antibody cocktail. Science. (2020) 369(6506):1010–4. <https://doi.org/10.1126/science.abd0827>.
2. Chen Y, Zhao X, Zhou H, Zhu H, Jiang S, Wang P. Broadly neutralizing antibodies to SARS-CoV-2 and other human coronaviruses. Nat Rev Immunol. (2023) 23(3):189–99. <https://doi.org/10.1038/s41577-022-00784-3>.
3. Martí D, Alsina M, Alemán C, Bertran O, Turon P, Torras J. Unravelling the molecular interactions between the SARS-CoV-2 RBD spike protein and various specific monoclonal antibodies. Biochimie . (2022) 193:90–102. <https://doi.org/10.1016/j.biochi.2021.10.013>.
4. Starr TN, Czudnochowski N, Liu Z, Zatta F, Park YJ, Addetia A, et al. SARS-CoV-2 RBD antibodies that maximize breadth and resistance to escape. Nature. (2021) 597(7874):97–102. <https://doi.org/10.1038/s41586-021-03807-6>.
5. Verkhivker GM, Di Paola L. Integrated Biophysical Modeling of the SARS-CoV-2 Spike Protein Binding and Allosteric Interactions with Antibodies. J Phys Chem B. (2021) 125(18):4596–619. <https://doi.org/10.1021/acs.jpcb.1c00395>.
6. Huang KYA, Chen X, Mohapatra A, Nguyen HTV, Schimanski L, Tan TK, et al. Structural basis for a conserved neutralization epitope on the receptor-binding domain of SARS-CoV-2. Nat Commun. (2023) 14(1). <https://doi.org/10.1038/s41467-023-35949-8>.
